# Clinical Study of Neurology Nursing on Cerebral Apoplexy Rehabilitation

**DOI:** 10.1515/tnsci-2019-0029

**Published:** 2019-07-22

**Authors:** Jie Chen, Shuangxi Li

**Affiliations:** 1 The first hospital of Lanzhou University, Lanzhou City, China

**Keywords:** neurology, cerebral apoplexy, rehabilitation nursing, key exchange algorithm

## Abstract

The incidence of cerebral apoplexy has been on the rise in recent years, and research on the treatment and care of this disease has also received much attention. Therefore, a clinical study of neurological care for cerebral apoplexy rehabilitation care was conducted. Firstly, the Diffie-Hellman key exchange algorithm is introduced, and then the Diffie-Hellman prediction model is established. The patient is taken as an example to carry out simulation test, and the prediction model is compared with the real data. The data of the network model training set, the verification set and the test set are obtained. Patients were divided into observation group and control group by random number table method. The evaluation indicators included the treatment efficiency and the quality of life of the two groups. The results showed that the effective rate of the observation group was higher than that of the control group, and the difference between the two groups was statistically significant (P < 0.05). The experiment proves that the clinical effect of neurological nursing on cerebral apoplexy rehabilitation nursing is positive, which can improve the limb movement and self-living ability of patients, improve the quality of life of patients and improve patient satisfaction. Clinical study of neurology nursing on cerebral apoplexy rehabilitation nursing is discussed in this paper, and the rehabilitation nursing method and formal standardized nursing process of this disease are standardized.

## 1 Introduction

Cerebral apoplexy is a clinically common cerebrovascular disease, mainly caused by atherosclerosis, vasospasm, abnormal blood circulation and ischemia and hypoxia of brain tissue [1]. Cerebral apoplexy patients mainly present with headache, dizziness, limb dysfunction, etc., which seriously affect the quality of life of patients. Cerebral apoplexy breaks out suddenly, the patient is not easy to recover, the course of disease is long, and the mortality rate is high [2]. At present, with the advancement of medicine, the mortality rate of this disease has been greatly improved, but most patients still have various sequelae. Therefore, how to effectively promote the rehabilitation of patients with hemiplegia and improve the quality of life of patients is the focus of treatment [3]. Good rehabilitation care plays an important role in the rehabilitation of cerebral apoplexy patients. In this context, a clinical study of neurological care for cerebral apoplexy rehabilitation care was initiated. To investigate the clinical effects of nursing on cerebral apoplexy rehabilitation, 82 cerebral apoplexy patients admitted between October 2015 and October 2016 were studied.

The rehabilitation nursing of cerebral apoplexy patients was studied based on Diffie-Hellman key exchange algorithm. Firstly, the large-scale analog-to-digital operation is performed on the PC by binary exponential decomposition method, and the application software is created. On the Internet/Intranet, the instant exchange of the server and the client endpoint is used, and the key exchange is completed according to the Diffie-Hellman rule. Then, the sample data set is established based on the total score of SCL-90 psychological scale test results, and the network model of the rehabilitation nursing condition prediction system of cerebral apoplexy patients based on Diffie-Hellman key exchange model is established with MATLAB. Thereby predicting the rehabilitation and care status of cerebral apoplexy patients.

Clinical research on cerebral apoplexy rehabilitation nursing was conducted based on neurological nursing. Firstly, the current research status of cerebral apoplexy rehabilitation nursing is expounded. Then the Diffie-Hellman key exchange algorithm is proposed, and then the Diffie-Hellman prediction model is established. The characteristics, implementation principles and flow of the algorithm and model are expounded. Finally, the algorithm and model are tested by the patient, and the experimental conclusion is obtained. The optimization and improvement strategy is proposed for the shortcomings of the algorithm.

## 2 Related Work

Related studies have pointed out that the patient’s central nervous system will exert a certain compensatory capacity and the nervous system may be reconstructed although cerebral apoplexy patients have varying degrees of neurological deficits. Therefore, the implementation of effective and feasible rehabilitation care for patients contributes to the recovery of motor function. In recent years, rehabilitation medicine has made great progress with the continuous advancement of medicine. Arith T pointed out that the implementation of scientific and reasonable rehabilitation

measures for patients can effectively reduce the disability and mortality of cerebral apoplexy patients, and play a positive role in the recovery of motor function [4]. Mohindra S and other studies found that cerebral apoplexy patients did not have effective rehabilitation during the rehabilitation period, resulting in limb muscle atrophy, joint deformation, and ultimately dysfunction [5]. After studying cerebral apoplexy rehabilitation care, Dohin B concluded that timely rehabilitation of cerebral apoplexy patients can effectively prevent the deterioration of motor dysfunction and help patients recover their functions as soon as possible [6]. Tachibana N and other recommendations for cerebral apoplexy rehabilitation care, that is, in rehabilitation care, the nursing staff first consult the patient actively, can improve the patient’s bad mood effectively, and promote the patient’s active cooperation with treatment and nursing measures [7]. Adachi T et al. found that the effective rate of neurological rehabilitation nursing intervention in cerebral apoplexy patients was 93.75%, which was significantly higher than that in the conventional nursing group (84.85%). The difference was statistically significant [8]. Chou KL and other early rehabilitation treatments can help patients calm down, and reduce anxiety, nervousness, irritability and other symptoms, then build confidence in rehabilitation as early as possible, and help patients with rehabilitation in a correct and positive attitude to achieve better treatment [9]. Block HS and other people believe that the nursing of the limbs of the nursing staff can promote the recovery of cerebral apoplexy effectively. The nursing staff should continue to massage the affected limbs 2 to 3 times a day. Each joint needs to be massaged 10 to 15 times. It can effectively avoid limb muscle wasting and deep vein thrombosis by massaging the affected limb [10].

In recent years, more and more scholars have paid attention to the clinical research of cerebral apoplexy rehabilitation nursing, and have also obtained rich research results. However, there are still few research results on neurological health care for cerebral apoplexy rehabilitation care, which is the direction that needs to continue in the future.

## 3 Information and methods

### 3.1 General Information

Considering the actual situation of our hospital, 82 stroke patients admitted to our hospital from October 2015 to October 2016 were selected as the analysis objects. The patients were randomly divided into observation group and control group, with 41 cases in each group. In the observation group, there were 22 males and 19 females respectively. The oldest ones were 82 years old, and the youngest was 45 years old. The average age was (68.27±10.59) years old, with 25 cases of cerebral hemorrhage, and 16 cases of cerebral infarction. In the group, there were 23 cases and 18 cases of males and females respectively. The oldest ones were 81 years old, and the youngest was 45 years old. The average age was (68.83±10.22) years old, with 24 cases of cerebral hemorrhage and 17 cases of cerebral infarction. There was no significant difference in the general information such as age and gender between the two groups, and there was no statistical significance (P>0.05), which had the value of comparison（Table 1）.

**Table 1:** Grouping of patients

Group	cerebral hemorrhage	cerebral infarction	Total
observation group	25	16	41
control group	24	17	41

### 3.2 Method

The two groups of patients used the general nursing method. On this basis, the observation group used other rehabilitation nursing methods:

1. psychological nursing, The care worker carefully observes the patient’s emotions, improves communication between patients, and comforts the patient at the fastest speed, such as fear. Worried about the problems, take the psychological counseling of the corresponding measures, change the bad mood of the patients as soon as possible, listen carefully and answer the questions pointed out to the patients and their families. This disease and treatment situation, actively encourage patients to help patients build confidence in rehabilitation.
2. Position Care: Post Care: The nursing staff guides the patient to complete the early rehabilitation training, which helps the patient to scientifically change his position. Based on the patient’s condition, he adjusts his position to prevent body contracture and pressure sore.
3. functional exercise: The nursing staff performs care according to the passive and active movements of the patient’s actual situation. In the early stage, the patient’s condition allows, the patient’s passive activities, mainly include massaging the patient’s muscles, and abduction, internal rotation, each limb joint Flexion and extension, etc. The large joints first, then small joints should gradually increase the amount of exercise, exercise 3 times a day, each exercise for 30 minutes is appropriate. The specific activity level and duration are determined based on the patient’s tolerance, such as permitting in the body, guiding the patient to practice, tightening and relaxing from simple joint flexion and extension muscles, starting from the recovery of muscle strength, guiding posture training, then practicing, balancing and walking, guiding patients to wash their faces, eating, etc.
4. swallowing dysfunction training: dysphagia care workers should first guide the patient to practice the pronunciation function, and then gradually practicing with chewing muscle function, performing mouth muscle training for the mouth, throat elevation, sucking and ingesting training. When the patient eats, he should take a semi-lying or sitting, and need to choose a certain viscosity instead of the food of the mouth.

### 3.3 Observation indicators

To evaluate the therapeutic effects of the two groups of patients, the specific conditions are as follows: basic rehabilitation: the degree of disability of the patient is 0. The degree of neurological impairment score is not less than 90%. Significant effect: The patient’s disability was 1 to 3 points, and the neurological impairment score decreased by 46% to 89%. Efficacy: The neurological deficit score was reduced by 18% to 45%. Invalid: The neurological impairment score decreased by less than 18%. Total effective rate = basic cure rate + significant cure rate + effective rate.

### 3.4 Statistical methods

The data above were summarized and combed by the SPSS18.0 tool, and the data was recorded by the chi-square test method. The data was counted by x2 test, and the measurement data was represented by the mean standard deviation. Using the t test, P < 0.05 represents a statistically significant difference between the two groups.

## 4 Research results

The effective rate of the observation group was significantly higher than that of the control group. The difference between the two groups was statistically significant, P<0.05, which had certain significance in statistics, as shown in Table 2.

**Table 2:** Comparison of therapeutic effects between observation group and control group [n (%)]

Group	Number of cases	invalid	effective	Markedly effective	effective rate
observation group	41	3	6	32	38（92.68）
control group	41	7	11	23	34（82.93）
x2					4.12
P					<0.05

Cerebral apoplexy has a sudden onset of symptoms, and is more likely to appear or die. It is a common condition in neurology. In recent years, its incidence has increased gradually, and research on the treatment and care of this disease has also received great attention. The central nervous system of these patients still has functional compensation and recombination functions after onset. It is hypothesized that rehabilitation measures should be taken early in the disease, which is expected to change functional impairment, and reconstruct new neural pathways, and restore related functions. Neurological rehabilitation nursing is to strengthen the psychological rehabilitation of patients on the basis of routine nursing, which can reduce the existence of negative emotions of patients, and guide patients to build confidence in rehabilitation, and actively cooperate with treatment. Strengthening rehabilitation exercise and nursing can enhance the intervention in position training, standing, walking, etc. It can avoid a series of complications such as joint deformity, muscle atrophy, body position and paralysis, thus changing the related motor function. The cultivation of daily life skills laid the foundation for their independent life. Using a range of tactile stimuli, pain stimuli, and acupuncture treatments can alter blood circulation and prevent negative events. Neurology rehabilitation care can significantly shorten the recovery time of patients, promote the rehabilitation of patients, and lay the foundation for patients to return to society as soon as possible. The central nervous system will exhibit compensatory ability to reconstruct the nervous system according to related research, although cerebral apoplexy patients have varying degrees of neurological deficit. Therefore, effective and feasible rehabilitation nursing measures are conducive to the recovery of the patient’s exercise capacity. In the analysis process of this subject, the effective rate of the observation group was higher than that of the control group, and the difference between the two groups of data was statistically significant (P<0.05). It can be learned from the above studies that neurological rehabilitation nursing intervention is beneficial to patients to recover their exercise ability more quickly. In recent years, rehabilitation medicine has also made certain progress with the rapid development of the medical industry. Rehabilitation care is an important part of rehabilitation medicine. Some scholars have proposed to adopt scientific and feasible rehabilitation nursing methods for patients. It is possible to minimize the possibility of disability and death in patients and to restore the patient’s athletic ability more quickly. A large number of cerebral apoplexy patients did not take effective rehabilitation care during the rehabilitation phase, resulting in muscle atrophy and joint deformation, leading to dysfunction. However, taking rehabilitation measures for cerebral apoplexy patients as soon as possible can prevent the deterioration of the patient’s motor function and enable the patient to resume exercise in time. In the process of rehabilitation nursing, the nursing staff must first take the initiative to take psychological counseling measures for the patients, which can change the negative emotions of the patients, and can make the patients cooperate with various treatment and nursing measures actively. According to relevant research, the therapeutic effect of neurological rehabilitation nursing intervention on cerebral apoplexy patients reached 93.75%, which was significantly higher than that of the conventional nursing group (84.85%). The difference was statistically significant (P < 0.05), consistent with the results. In summary, the clinical rehabilitation and nursing effect of cerebral apoplexy patients in neurological nursing is better, which is conducive to the rehabilitation of patients and significantly improves the quality of life of patients, which is worthy of further adoption.

Stroke is a common disease in neurology because of its sudden onset and high risk of disability or death. In this study, the traditional nursing mode was adopted in the control group, while the rehabilitation nursing in the experimental group was carried out in the Department of neurology. The results showed that the clinical satisfaction of the experimental group was higher than that of the control group (P < 0.05); there was no significant difference in limb movement, independent living ability and quality of life before nursing; the limb movement, independent living ability and quality of life of the experimental group were better than those of the control group (P < 0.05); the rehabilitation effect of the experimental group was higher than that of the control group (P < 0.05). To sum up, the clinical effect of Neurology nursing on stroke rehabilitation nursing is affirmative. It can improve patients’limb movement, independent living ability, promote their quality of life, and improve patients’ satisfaction.The method proposed in this paper is superior, but it is more complicated than the prediction. The expression of stroke pathology by equation is only approximate. To achieve the goal of reducing errors, the calculation parameters need to be screened and corrected in depth.

## 5 Conclusion

Cerebral apoplexy is a common neurological disease with a high risk of sudden onset, disability or death. Based on the Diffie-Hellman key exchange algorithm, the clinical nursing of cerebral apoplexy rehabilitation nursing for neurological nursing was carried out. Firstly, the large-scale modulo operation is performed on the PC by the binary exponential decomposition method, and the key exchange is completed according to the Diffie-Hellman rule. Then the network model of the patient rehabilitation nursing condition prediction system based on Diffie-Hellman key exchange model is established with MATLAB, thereby realizing the prediction of the rehabilitation and nursing status of cerebral apoplexy patients. After the sample training, the final output of Diffie-Hellman prediction model is output. The error between the predicted value and the measured value is only 3.04%, which is better than expected. In this study, the control model of the control group was used as the traditional model. The experimental group performed Neurological Rehabilitation Nursing. The results showed that the clinical satisfaction of the experimental group was higher than that of the control group (P<0.05). There was no significant difference in limb movement, self-living ability and quality of life before treatment. After exercise, the limb exercise capacity, independent living ability and quality of life of the experimental group were better than the control group (P<0.05). The rehabilitation effect of the experimental group was better than that of the control group, P<0.05. In summary, the clinical effect of neurological care on cerebral apoplexy rehabilitation nursing is positive, which can improve the patient’s limb movement and independent living ability, and improve the patient’s quality of life and satisfaction. Although the proposed algorithm and the constructed model are superior in accuracy and stability, the actual situation is much more complicated than the prediction. Applying the equation to express the pathology of cerebral apoplexy is only an approximation. The parameters are implemented in an intensive screening and calibration.
